# Extra-follicular cutaneous manifestations of frontal fibrosing alopecia^☆^

**DOI:** 10.1016/j.abd.2024.01.003

**Published:** 2024-08-30

**Authors:** Aline Donati, Isabelle I. Hue Wu

**Affiliations:** a Trichology Outpatient Clinic, Hospital do Servidor Público Municipal de São Paulo, São Paulo, SP, Brazil; b Laser Outpatient Clinic, Hospital das Clínicas, Faculty of Medicine, Universidade de São Paulo, São Paulo, SP, Brazil

**Keywords:** Alopecia, Lichen planus, Skin

## Abstract

Frontal fibrosing alopecia (FFA) is an inflammatory cicatricial alopecia, which is considered to be a variant of lichen planopilaris. In addition to follicular changes, FFA often presents with associated cutaneous manifestations in most patients, including lichen planus pigmentosus, implantation line hypochromia and facial papules. The objective of the present article is to provide a detailed overview of the non-follicular cutaneous clinical manifestations of FFA and discuss their impact on the diagnosis and treatment of patients with this condition.

## Introduction

Frontal fibrosing alopecia (FFA) is an inflammatory and cicatricial alopecia histopathologically identical to lichen planopilaris,^1^, ^2^ which is considered a clinical variant of this disease.^3^ It was first described in 1994 in Australia^4^ and its frequency has been increasing globally in recent decades.^5^ It predominantly affects adult women,^6^ and with no reported cases in children to date.^7^ There is no clear predilection for ethnicity, but most studies involve Caucasian individuals^8^ due to the prevalence of this ethnicity in the countries where the research was conducted. Although initially associated with the postmenopausal period, the proportion of premenopausal women has been progressively increasing in series published in recent years,^9^, ^10^ partly due to the earlier identification of the disease.

The etiopathogenesis of FFA remains not fully understood. Its recent description, combined with the growing prevalence of the disease in several countries, reinforces the hypothesis of an environmental trigger acting on genetically predisposed individuals.^11^ In this sense, the association between the disease and the frequent use of facial cosmetics found in case-control studies in recent years has garnered significant scientific attention, even though it did not prove a causal relationship.^12^, ^15^ Several studies investigating skin reactivity to substances in facial cosmetics in FFA patients demonstrated a higher prevalence of allergic contact dermatitis in this population,^16^ with special emphasis on fragrances and preservatives.^12^, ^17^, ^18^

The disease classically affects the anterior implantation line of the scalp, leading to a slowly progressive increase in the size of the forehead.^8^ The preferential and/or initial involvement of the vellus follicles in this region helps differenctiate FFA from other band alopecias that can affect the anterior border of the scalp, making dermoscopic examination a crucial tool for the early diagnosis of the disease.^19^, ^20^ The anterior border involvement shows three clinically disctinct patterns thar appear to correlate with different disease prognoses: Patterns 1 (regular) and 2 (irregular) show the typical regression of the implantation line and consequent increase in the forehead, whereas in pattern 3 (pseudo-fringe) the alopecia occurs just behind the implantation line, preserving a strip of terminal hairs that simulate the fringe sign seen in traction alopecia.^21^

In addition to the anterior border of the scalp, the eyebrows are also affected in most patients,^8^ with madarosis being a key clinical sign for suspecting the disease. Other areas of the scalp, facial hair and, in short, any hair on the body can also be affected by the disease in varying proportions across different studies to date.^6^, ^8^, ^22^

Although much less highlighted in publications about the disease, FFA can also affect other skin structures. The non-follicular skin changes associated with the disease to date can be didactically classified into two types: (1) Changes in the dermoepidermal junction and (2) Cutaneous atrophy.

Some studies suggest that the extra-follicular cutaneous involvement may precede alopecia and recognizing these changes could aid in earlier diagnosis. Moreover, other therapeutic possibilities may be indicated, viewing the disease as a disorder that is not only follicular and inflammatory but also afeccting other skin structures. The aim of this review is to detail the non-follicular cutaneous clinical manifestations of FFA and discuss their impact on the diagnosis and treatment of patients with the disease.

## Changes in the dermoepidermal junction

Cutaneous manifestations secondary to involvement of the dermoepidermal junction can generate four main clinical presentations: (1.1) Hyperchromic macules or melasma-like lesions; (1.2) Achromic macules or vitiligo-like lesions; (1.3) Hypochromia at the implantation line and (1.4) Erythematous macules or rosacea-like lesions.

The dermo-epidermal junction (DEJ) is the location with the highest concentration of melanocytes and lesions in this region can affect the activity of these cells. Depending on the number and activity of melanocytes in the basal layer of the epidermis and the effect of inflammation on them, three colors may appear clinically on the skin. When the amount of pigment produced in the basal layer of the epidermis is high, inflammation of the DEJ generates significant pigment spillage and, consequently, hyperchromic macules develop there. When this inflammation generates a reduction in the number and/or activity of local melanocytes, hypo/achromic changes will occur. In cases where inflammation affects fair skin, macular or reticular erythema may be observed. Erythema may or may not precede the other described dyschromias.^23^

### Hyperchromic macules or melasma-like lesions

Among hyperchromic changes, the most important is lichen planus pigmentosus (LPP);^9^ however, lentiginous lesions have also been described.^24^

### Lichen planus pigmentosus

#### Clinical description

**Fig. 1:**
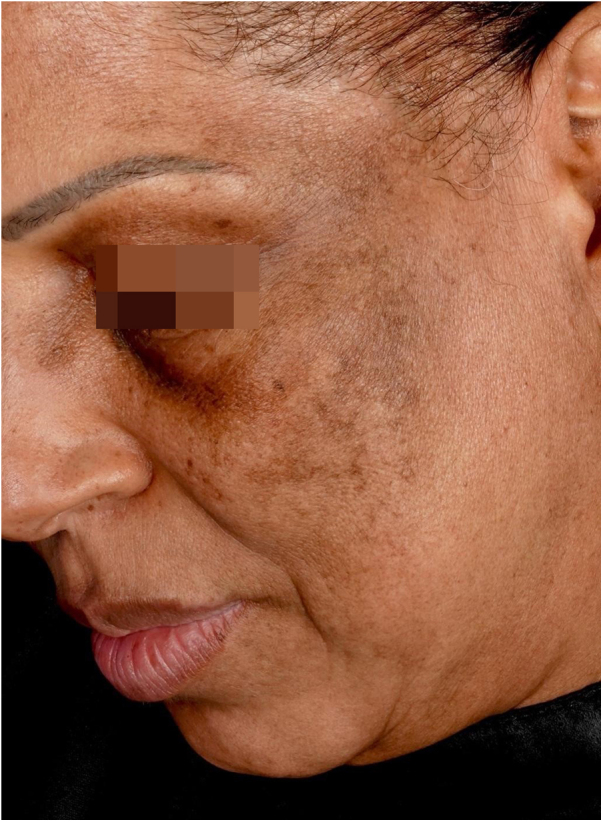
Reticular brownish macules in the malar, zygomatic and periorbital regions of a patient diagnosed with FFA.

Reticular or diffuse macules ranging in color from brown to black, gray and blue (Fig. 1). LPP associated with FFA can vary widely in extent and is most commonly seen on the face but can also be found in other areas of the body, such as the neck, anterior chest and upper limbs.^25^ It predominantly affects photo-exposed areas, clinically resembling melasma, but it can also affect flexural sites.^9^ Involvement of the upper eyelids is highly suggestive of this diagnosis and helps to differentiate it from melasma.^26^

The dermoscopy features of LPP associated with FFA was described in 2016 and includes varied patterns such as pseudo-network, blue-gray dots arranged in a circular or speckled conformation, rhomboidal structures, asymmetric pigmentation in the follicular openings and a dotted pattern, with the latter corresponding to the involvement of the ostia of eccrine ducts.^27^ Vellus hairs may or may not be reduced at the site, demonstrating the dissociation between follicular involvement and dermoepidermal junction inflammation.

The similarity between the mechanism of LPP hyperchromia associated to FFA with dermal melasma and post-inflammatory hyperpigmentation means that these entities are very similar clinically, dermoscopically and histopathologically. In practice, the diagnosis of this condition only occurs in patients who have follicular involvement of FFA.

#### History

The association of FFA with LPP was first described by Dlova in South Africa in 2013.^9^ In this initial series of cases, 54.5% of the 44 patients described with FFA had hyperchromic macules on photoexposed areas, where histopathological analysis confirmed LPP. It is worth noting that the appearance of skin lesions preceded alopecia in all patients, with an average interval of 14 months.^9^ Another interesting fact is that in the group of patients who showed an association of FFA and LPP, the majority were pre-menopausal women, in contrast to all the studies published until then.^28^ According to the author of the aforementioned study, the high frequency of traction hairstyles in this Afro-descendant population might explain the triggering of the follicular inflammatory process earlier.^9^

#### Epidemiology and prevalence

The association between FFA and LPP is more frequent in individuals with higher phototypes^29^ due to the greater amount of pigment in this group. This means that the prevalence of this association varies greatly depending on the predominant ethnicity in the region where the study is published. While Dlova described the association of FFA with LPP in 54% of their 44 patients in South Africa,^9^ the prevalence was 40% in a study with 20 patients with FFA in Morocco^10^ and 20.7% in a study with 58 patients in Thailand.^24^ In contrast, in Argentina^30^ and Spain,^14^ where the majority of patients have lower phototypes, two large case series showed this association in only 4.5% and 3.1% of the cases, respectively. In Brazil, it is likely that the frequency of LPP associated with FFA varies with the type of racial miscegenation in different regions of the country. In São Paulo, a case series found LPP concomitantly in 14% of the 114 studied patients with FFA.^31^

The frequency of LPP associated with FFA seems to be more frequent in women^14^ and most studies of FFA in men do not mention this finding in their results. A single study observed a correlation between a greater extension of alopecia and the presence of facial lentiginous macules (OR = 5.87; 95% CI 1.15–29.9; p = 0.033) but did not find a correlation between the extension of alopecia and LPP.^24^

In addition to the clear predisposition of patients with FFA with higher phototypes to have LPP, there is a curious association between these skin lesions and the diagnosis of hair disease at younger ages and, consequently, before menopause.^32^ In addition to the data published by Dlova, which showed that 64% of their patients with FFA and LPP were premenopausal,^9^ 65% of the cases in the Morocco series were not in menopause either^10^ and neither were 37.6% in the series from Thailand.^24^ In contrast, a systematic review of 932 predominantly white patients showed that 84.1% of FFA cases were diagnosed after menopause.^8^

An interesting study carried out in Miami showed that the Caucasian population of Hispanic origin presents the association of LPP with FFA more frequently than non-Hispanic Caucasians (22% vs. 6%, p = 0.025).^25^ In this same article, there is a higher prevalence of pre-menopausal women in the group of Hispanic descendants (60% vs. 37%; p = 0.039) and also a higher frequency of LPP in pre-menopausal women (23% vs. 9 %; p not significant).^25^

#### Physiopathogenesis and histopathology

The etiopathogenesis of LPP is not completely known, but it is possible that photosensitizing chemical substances are involved.^33^ Similarly, LPP associated with FFA may also be related to products applied topically to the skin, corroborating the association found between FFA and the frequent use of creams, including sunscreens.^12^, ^13^

**Fig. 2:**
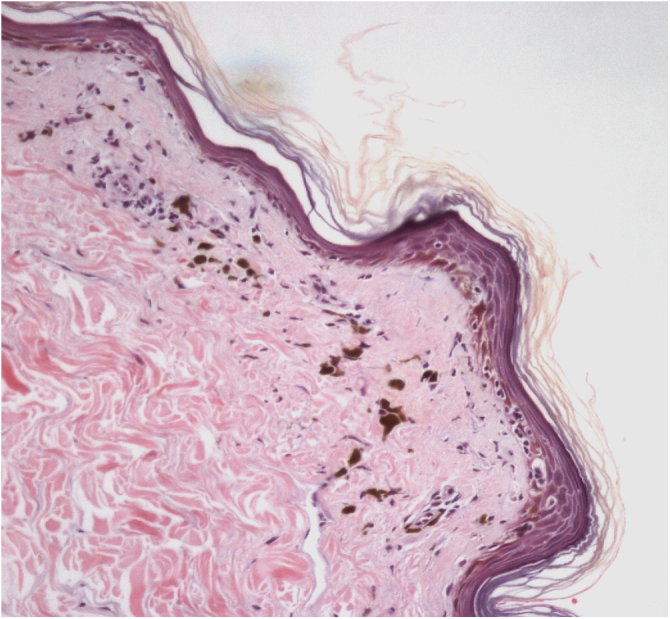
Histopathology of LPP lesion showing lichenoid interface dermatitis and incontinentia pigmenti (Hematoxylin & eosin, ×40).

The histopathological examination of classic LPP in the initial phase shows dermal-epidermal lichenoid interface dermatitis with varying degrees of pigment spillage (Fig. 2).^33^ The dermal interface with the follicular epithelium may also be affected, with perifollicular inflammatory infiltration and changes in the basal layer of the infundibulum in some cases.^33^ In a specific study of histopathological findings of LPP associated with FFA, the presence of inflammation in sebaceous and sweat glands was also observed.^31^

In the residual phase of the disease, only pigment spillage is observed beneath the generally thinned epidermis. At this stage, the histology of LPP is identical to that of any post-inflammatory hyperpigmentation and it may be difficult to differentiate it from melasma with a dermal component.

#### Temporality

A very interesting observation published by Dlova in their first description of the association of LPP and FFA is that hyperchromic lesions often precede alopecia.^9^ In that study, the interval between cutaneous and hair disease varied from six to 36 months, with an average of 14 months. Although the other studies on LPP associated with FFA do not usually mention the temporal relationship between the two clinical manifestations, the fact that case series in populations with higher phototypes show a younger mean age at diagnosis^9^, ^10^, ^24^ can also be considered an indication that the dermoepidermal junction dermatitis precedes follicular involvement.

This fact has very relevant implications for the understanding of the disease. Firstly, it reinforces the hypothesis that FFA is triggered by topical substances on the skin.^12^, ^13^ Secondly, it suggests that a large proportion of Caucasian patients with FFA may have a long period of silent disease before diagnosis, because there is insufficient pigment spillage to generate clinically visible lesions in the areas with inflammation of the dermoepidermal junction. Therefore, in addition to the fact that this is a disease with a slow evolution,^6^ any study that aims to investigate the possible causal factors of FFA should cover contact with topical medicines that occurred many years before the diagnosis and not after the diagnosis was established.

#### Treatment

There is no international consensus on the treatment of lichen planus pigmentosus, but understanding the pathophysiology of the disease can help manage affected patients. In general terms, the treatment must consider the active phase of the interface dermatitis. In the acute phase, the treatment should focus on controlling inflammation, whereas in the residual phase, treatment aims to remove the melanin pigment from the dermis. For the inflammatory phase, any medication useful for other dermoepidermal junction dermatitis, such as cutaneous lichen planus, lupus erythematosus and dermatomyositis, can be used. In this sense, the use of methotrexate seems promising, although there are no prospective studies to date that have proven its effectiveness.

Hydroxychloroquine, despite being potentially effective as an anti-inflammatory drug, can lead to worsening of pre-existing pigmentation^34^ and it is prudent to avoid its use in patients with FFA and hyperchromic lesions. As this is a chronic and recurrent disease,^33^ the use of systemic corticosteroids must be extremely rare. Similar to what is suggested for classic LPP, the use of oral isotretinoin could have an anti-inflammatory and lesion-lightening benefit at a dose of 20 mg/d,^35^ but the experience of one of the authors shows this drug not to be effective in patients with LPP associated with FFA.

**Fig. 3:**
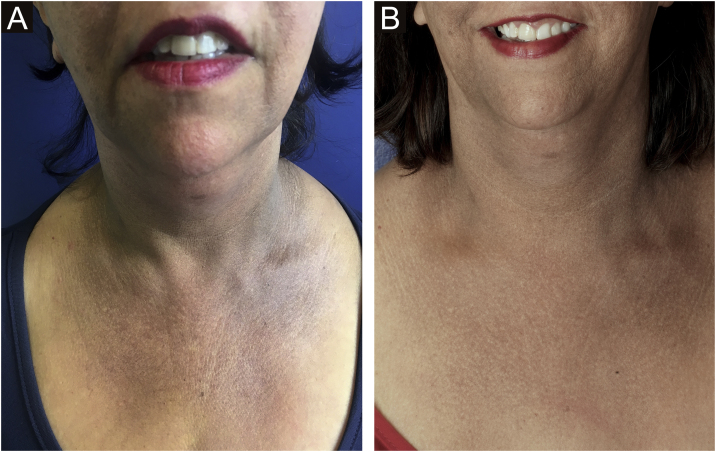
LPP on the anterior chest of a patient with FFA before treatment with Nd:YAG Q-Switched Laser (A) and after laser treatment performed (B). Source: photos provided by Dra. Susana Wu.

Aimed to lighten the residual lesions of classic LPP, there are reports on the use of topical formulations containing hydroquinone, retinoid, tacrolimus, and topical corticosteroids.^36^ Considering that LPP associated with FFA and FFA itself can be caused by a topical agent, the authors suggest avoiding the use of topical formulations in these cases. Among the physical procedures capable of skin lightening, a case report of a patient submitted to dermabrasion showed significant improvement of the hyperchromia.^37^ Even after a slight recurrence of the lesions one year later, the patient remained satisfied. Recently, a study was published showing significant improvement in the size and intensity of pigmentation in ten of 13 patients treated with Q-switched Nd:YAG laser at low fluence (Fig. 3).^38^ There is no post-treatment follow-up data for these patients, and it is possible that pigmentation may return if inflammation of the dermoepidermal junction is not controlled. With the exception of this latter laser study, all other treatments have been described only in case reports or retrospective studies.

In recurrent cases, it is suggested to investigate the likely trigger of the process and discontinue it. The use of any topical substance that generates inflammatory symptoms or signs on the skin should be discouraged in these patients. The indiscriminate suspension of all topical products may be suggested in cases with active inflammation that is refractory to current treatments.

#### Relevance

The frequent association of LPP with FFA should keep doctors alert in all cases of melasma-like lesions in adult women for the possibility of early FFA. According to a study published in 2018, LPP lesions place Spanish women at 5.14 times more risk of having the disease.^14^ Whenever LPP is suspected, careful dermoscopy of the entire implantation line must be performed and a biopsy must be taken from the area with the absence of vellus hairs. Although the dermoepidermal junction can be affected before the follicle, the diagnosis of FFA can only be made when alopecia has already developed.

The involvement of the dermoepidermal junction expands the understanding of the disease. FFA changes from a purely follicular condition, similar to lichen planopilaris, to a disease that affects the dermal-epidermal interface, bringing it closer to cutaneous lichen planus, lupus erythematosus and dermatomyositis. Effective treatments for these other dermoepidermal junction dermatitis, such as methotrexate, become potentially useful in these patients. Furthermore, the fact that these lesions precede hair loss has important implications for understanding the etiolgy of the disease. This suggests that the disease can begin in the epidermis and it may take years for follicular involvement to occur or become evident. On one hand, this reinforces the potential role of topical agents in triggering of the FFA process.^12^, ^13^ On the other hand, it implies that contact with the likely trigger must have occurred long before the occurrence of alopecia and its diagnosis. Therefore, the more frequent use of creams found in FFA patients in case-control studies might be a consequence of the facial sking changes caused by the disease years earlier, rather than the cause of the disease itself.^39^

### Achromic macules or vitiligo-like lesions

#### Clinical description

**Fig. 4:**
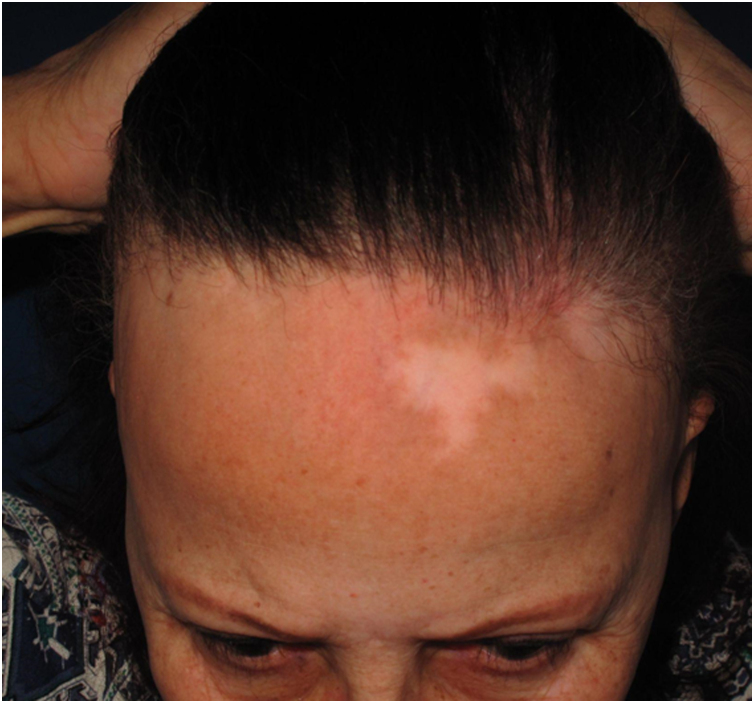
Achromic macules on the region of the hair implantation line in a patient diagnosed with FFA.

Achromic macules with well-defined limits may present as a single lesion of variable extent or multiple small achromic areas amidst normal or hyperchromic skin. The anterior border of the scalp is the most frequently affected area (Fig. 4), but it is likely that all areas affected by LPP may also show vitiligoid lesions, since they present the same histopathological changes.

#### History

In 2014, an article was published demonstrating that dermoepidermal junction dermatitis may be responsible for achromic vitiligoid lesions in patients with FFA, in a similar way to what is observed in subacute vitiligoid lupus erythematosus or in the melasma-like variant of actinic lichen planus. In this case report, the location of the lesions on the anterior border of the scalp and neck, in addition to histopathological changes in the dermoepidermal junction, indicated that the same process responsible for the hyperchromic lesions published as LPP associated with FFA in the previous year might, more rarely, generate clinically achromic, rather than hyperchromic lesions.^40^

There are some reports of vitiligo in patients with FFA. In these cases, histopathology is essential to exclude the possibility of vitiligoid lesions resulting from dermoepidermal junction dermatitis. Even in these cases where two different associated diagnoses are suggested, the hypothesis of a common pathophysiological pathway for the two diseases involving cytotoxicity of CD8 + T lymphocytes in melanocytes and keratinocytes is considered.^41^, ^42^

#### Epidemiology and prevalence

The frequency of vitiligoid lesions associated with FFA is probably rare. Even if misdiagnosed as vitiligo, the association of the two diseases was found in only 1.1% of cases in a review of 932 patients,^8^ 2.4% in a large Spanish case-control study^14^ and 2.5% in the largest French-German series published to date.^43^ All reports showed that the prevalence of vitiligo in patients with FFA was very close to that found in the general population, which is 0.5%–2.0%.^44^

Achromic lesions associated with FFA can occur in patients with any phototype, but are more evident in more pigmented skin.

#### Physiopathogenesis and histopathology

The main histopathological finding of vitiligoid lesions associated with FFA is the presence of changes in the dermoepidermal junction, including some degree of pigment spillage.^40^ Unlike LPP, however, the amount of pigment spillage is generally small. It is likely that the physiopathogenesis of this condition is similar to that of LPP, with the participation of topical photosensitizing substances.

#### Temporality

The case report that described vitiligoid lesions associated with FFA stated that they appeared years after the diagnosis of alopecia.^40^ The fact that achromic lesions appeared before the diagnosis of FFA in some reports cannot be considered sufficient to exclude the possibility of vitiligoid lesions associated with FFA. Similar to what occurs with LPP, epidermal interface dermatitis may precede the follicular involvement of FFA by years.^9^

#### Treatment

There are no reports on the treatment of vitiligoid lesions associated with FFA. Considering its probable inflammatory etiology, the same treatments proposed for vitiligo should be considered.^45^ Due to the possible role of topical substances and photosensitivity in the etiopathogenesis of lesions, the use of topical and photosensitizing substances should preferably be avoided.

#### Relevance

Vitiligoid lesions, similar to LPP, expand the understanding of FFA as a disease of the dermoepidermal junction and not just follicular in nature.

### Hypochromia of the hair implantation line

#### Clinical description

**Fig. 5:**
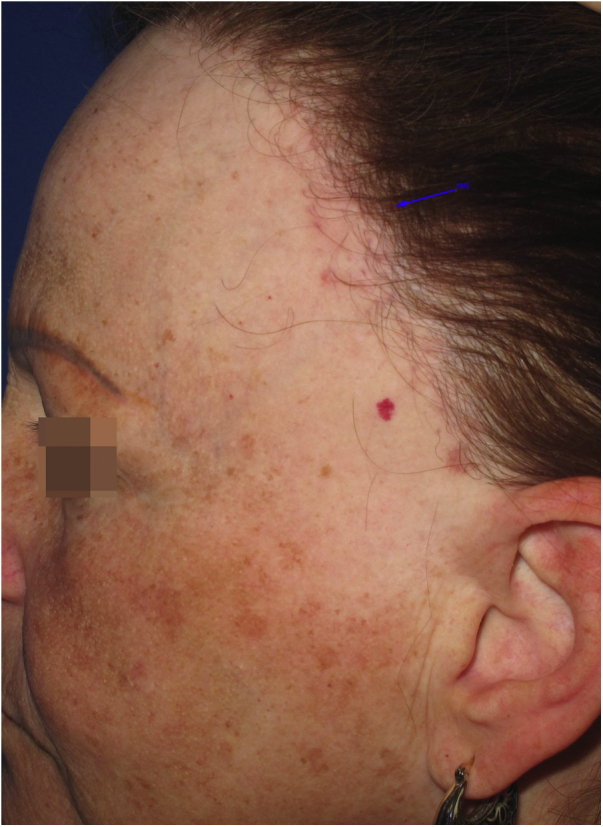
Ill-defined hypochromic macule on the region of the hair implantation line in a patient with FFA.

This constitutes a poorly delimited hypochromic area in the region of the hair implantation line affected by FFA (Fig. 5). The region is more visible when the facial skin has a high degree of pigmentation and photodamage. In patients with fair skin and little photodamage, the use of Wood’s lamp can help highlight hypochromia.^46^ It is generally associated with skin thinning observed clinically as smooth skin^10^ with easily seen larger blood vessels.^47^

#### History

In his first description of the disease in 1994, Kossard describes hypochromia of the hair implantation line contrasting with the skin of the forehead as a clinical characteristic of this condition.^4^ At the time, the difference in color between the affected border of the scalp and the photodamaged skin of the face was justified as a result of the photoprotection normally provided by the hair. Years later, a study showed that this hypochromia is not the result of lack of sun exposure, but rather a reduction in the number of melanocytes in the area.^46^

#### Epidemiology and prevalence

There are no studies on the frequency of this finding in patients with FFA, but in practice, it is not an uncommon finding. In a study on hypopigmentation in the eyebrow region, 84% of the 20 described patients also had hairline hypopigmentation, corroborating the clinical impression that this is a frequent alteration. All patients in this study had a high phototype, but it is likely that this fact is just a facilitator when observing this condition.^48^

#### Physiopathogenesis and histopathology

A study with scalp biopsies published in 2017 showed a reduction in the number of melanocytes in five cases of FFA compared with four cases of lichen planopilaris and ten control patients.^46^ In addition to the small sample size, a probable normal variation of this parameter in different regions of the scalp must be considered.

This same study also suggests lower epidermal thickness in this small series of FFA patients,^46^ but a subsequent study using confocal microscopy and comparison of the same region of the scalp showed discordant results.^49^ Considering that melanocytes are located in the basal layer of the epidermis, their reduction may also be an indication of involvement of the dermoepidermal junction. Recently, a Brazilian study identified a reduction in the number of melanocytes in the upper region of the hair follicle in a patient with FFA, but without a control group.^50^

#### Temporality

There is no data on the temporal relationship between hair implantation line hypochromia and alopecia.

#### Treatment

No treatment has been suggested to date.

#### Relevance

Hypochromia of the affected border of the scalp is probably part of the manifestations of dermoepidermal junction dermatitis associated with FFA. Its identification can be useful to differentiate it from other alopecias of the anterior border of the scalp, such as a high forehead or alopecia areata ophiasic.

### Erythematous macules or rosacea-like lesions

#### Clinical description

**Fig. 6:**
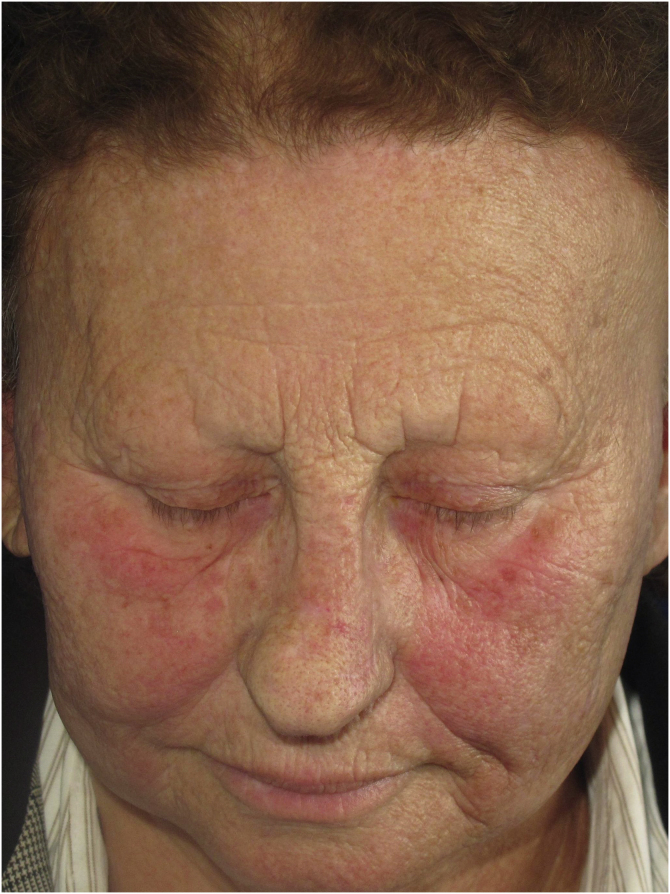
Erythema and telangiectasias on the malar, zygomatic and peri-palpebral regions in a patient with FFA.

Diffuse or poorly defined reticulated erythema, clinically similar to erythematous-telangiectatic rosacea (Fig. 6) can be observed, generally located in the center of the face. It may eventually occur in the eyebrow region or affect the entire face. Exacerbation of the lesion by triggering factors such as heat and consumption of alcoholic beverages is characteristic, analogous to what is observed in rosacea. Greater skin sensitivity to different contacts is often associated. There are no clear criteria that define whether these changes are just rosacea-like or whether the patient actually has rosacea as a skin disease associated with FFA. Interface dermatitis on histopathology can be considered a criterion that is present in FFA with rosacea-like changes but not in rosacea.

#### History

The description of erythematous lesions associated with FFA was published in 2015 in a Spanish study on facial lesions associated with FFA. Seven patients were described in this article, with a previous diagnosis of rosacea due to facial erythema associated with a burning sensation at the site, but in which histopathology showed dermatitis at the dermoepidermal junction.^23^

#### Epidemiology and prevalence

There are no data on the prevalence of rosacea-like changes in patients with FFA. In a Spanish study with 103 patients, rosacea was considered the dermatological comorbidity most frequently associated with FFA.^51^ While the general Spanish population has a 10% frequency of rosacea, the group of patients with FFA had this diagnosis in 34% of cases. It is worth noting that the majority of these cases were classified as erythematous-telangiectatic rosacea,^51^ a form of the disease that is clinically indistinguishable from the erythematous lesions associated with FFA. It is questioned whether some of these cases described as rosacea could, in fact, be erythematous lesions associated with FFA, since biopsy of erythematous lesions on the face is rarely performed.

All cases of FFA with erythematous lesions described in 2015 were in Caucasian individuals.^23^ Patient phototype can make it easier or more difficult to observe these color changes, with erythema being easier to observe in patients with a low phototype.

#### Physiopathogenesis and histopathology

The physiopathological mechanism of rosaceiform changes seems to be similar to that of other clinical manifestations resulting from dermoepidermal junction dermatitis associated with FFA. Similarly, the histopathological findings of erythematous lesions also include epidermal thinning, interfollicular lichenoid infiltrate, keratinocyte apoptosis and incontinentia pigmenti in the dermis.^23^ The presence of interface dermatitis can help differentiate it from erythematous-telangiectatic rosacea, which shows a predominance of perivascular lymphohistiocytic inflammatory infiltrate and dilation of superficial vessels on histopathology.^52^ There is a hypothesis that erythematous lesions may be the final clinical manifestation of dermoepidermal junction dermatitis in patients with low phototype or the early phase of this dermatitis that will culminate in LPP-type hyperchromic lesions in patients with a higher degree of epidermal pigmentation.^23^

#### Temporality

There are no published data on the temporal relationship between the appearance of rosacea-like changes and alopecia onset. According to the authors experience, this manifestation usually precedes alopecia by many years and is generally diagnosed and treated as rosacea for a long time.

#### Treatment

There is no description of the treatment of rosacea-like changes associated with FFA. If there is histopathological proof of dermoepidermal junction dermatitis, the same treatment used in LPP must be followed. As in most cases a biopsy is not performed on the face, the presence of rosacea-like manifestations is initially approached in the same way as classic rosacea.^53^ Doxycycline and oral isotretinoin are interesting options in these cases, as they may also be useful to control follicular inflammation and facial papules when associated. Whenever possible, potential crisis triggers should be avoided, as well as all potential skin irritants. Intense pulsed light and Nd:YAG laser can be used to improve the aesthetic appearance of erythema and telangiectasias.

#### Relevance

One must always remember that rosacea in adult women can be the initial manifestation of FFA that is still subclinical. According to a study published in 2018, rosacea-like lesions give Spanish women almost twice the risk of having the disease.^14^ It is important to consider FFA in every woman complaining of rosaceiform lesions and to perform dermoscopy of the hair implantation line looking for areas without vellus hairs. Histopathology demonstrating the presence of cicatricial inflammatory alopecia at the border of the scalp is necessary to make the diagnosis of FFA.

## Skin atrophy

Skin thinning is the least studied skin change associated with FFA. Cutaneous atrophy manifests itself clinically in two ways: (2.1) depression of the frontal veins and (2.2) facial papules.

### Depression of the frontal veins

#### Clinical description

Sinking of the skin is observed over the course of the frontal veins. The skin on the forehead is thinner and more pleated than usual, resembling aged skin.

#### History

**Fig. 7:**
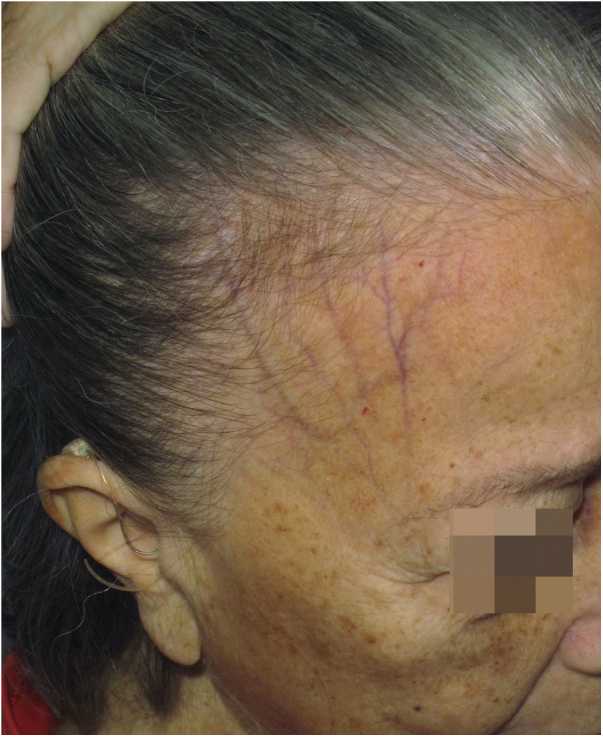
Cutaneous atrophy and depression of the forehead veins in a patient with FFA.

Although clinically visible in images published since the first reports of the disease,^4^, ^54^ skin atrophy was only postulated as a clinical manifestation of FFA in 2015 in an article that highlighted the depression of the forehead veins as a clinical sign of the disease (Fig. 7).^47^ It is understandable that the advanced age of patients and the frequent use of topical corticosteroids in the region delayed the identification of this clinical characteristic of the disease, which is why the authors who described this sign highlighted that four of the 11 reported patients had not used topical corticosteroids and the remaining noticed the depression before using this medication.

#### Epidemiology and prevalence

Few FFA case series mention depression of the frontal veins in their results. The sign was initially described in Caucasian patients,^47^ but a case series of 56 Thai patients also observed this finding in 7.1% of the patients.^32^ There are no data on the possible correlation between the frontal vein depression sign and the patients age, although it seems obvious this sign must be more frequent in older patients. A single study in men with FFA reported the finding in 12.8% of 39 examined Spanish patients.^55^ In 2017, a Spanish study on the different types of frontal implantation line involvement found that depression of the frontal veins was observed more frequently in type 1 (11%) than in type 2 (7.3%) and was not observed in type 3.^21^

#### Physiopathogenesis and histopathology

The mechanism that generates skin atrophy in patients with FFA is not known, nor exactly which layer of the skin is affected.

**Fig. 8:**
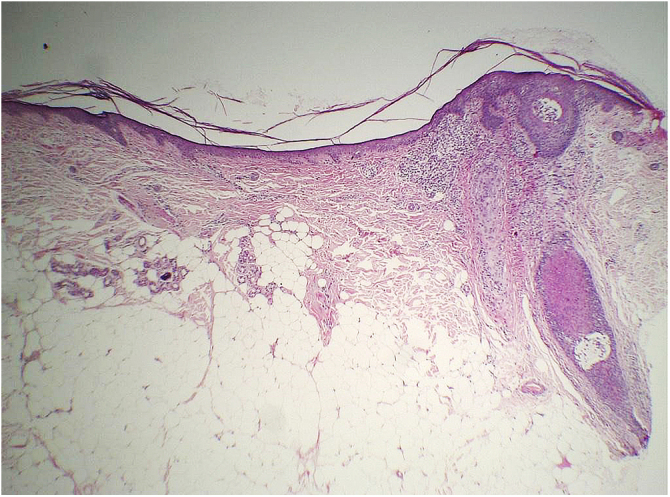
Anatomopathological examination of the skin showing significant dermal atrophy (Hematoxylin & eosin ×40).

Histopathology of two patients in the initial description of this sign showed significant atrophy of the dermis (Fig. 8), but no objective parameter of this change was provided to prove this statement.^47^

Considering that the normal range of forehead dermal thickness for the general population is not known, only a comparative study with a control group matched for the main variables that can influence dermal thickness (forehead region, age, phototype and photodamage) would be capable of verifying whether there is a decrease in dermal thickness and/or another layer of the skin related to the disease.

As the collection of material for histopathological examination would be inadvisable as a methodology for studying this facial region, the ideal would be non-invasive imaging methods to assess the thickness of the different skin layers. In this sense, to date, there is a study using confocal microscopy that demonstrated there is no epidermal thinning related to FFA, but due to the shallow image depth on confocal microscopy, it was not possible to assess dermal or hypodermal thickness.^49^ There is also a study using optical coherence tomography in patients with FFA, but it did not evaluate skin thickness.^56^

Future controlled studies with imaging devices capable of accurately measuring distances to the hypodermis are necessary to prove whether there is dermal and/or hypodermal atrophy related to the disease.

#### Temporality

According to the initial description in the article, the frontal vein depression sign occurs shortly after the onset of alopecia.^47^

#### Treatment

No treatment is suggested to treat cutaneous atrophy, but it is understood that the use of topical or injectable corticosteroids may worsen the condition, and these medications should be used with caution in patients with FFA.^47^ It is not known whether reversing this atrophy would have any impact on the alopecia evolution.

#### Relevance

Depression of the frontal veins was the first sign that suggested there was atrophy caused by the disease. Understanding that FFA is an atrophying disease implies the need for extreme caution when using topical or injectable corticosteroids in these patients.

### Papules on the face

#### Clinical description

Small follicular, monomorphic, non-inflammatory papules, with a smooth surface and ill-defined borders are observed. They may occasionally have delicate follicular spicules, similar to keratosis pilaris.^57^ They can affect practically any part of the face, but predominate on the temporal regions. The irregular texture caused by the papules eventually resembles skin with multiple acne scars.

#### History

Papules associated with follicular spicules were initially described in 2007, and were compared to Piccardi-Lassueur-Graham-Little syndrome.^57^ Later, in 2011, papules with a smooth surface were described, which are the most frequently found.^58^

#### Epidemiology and prevalence

The prevalence of papules on the face varies greatly among the numerous case series of the disease published to date. In the large Spanish series of 355 patients published in 2014, 14% of the cases had papules on the face, more commonly in men (33%) than in women (10 %–14 %). A more recent Spanish study with 75 patients showed that the prevalence of papules also varied with age, and was more frequent in patients under 55 years of age (85.7%), when compared to patients over 55 years old (22.6%; p = 0.002).^59^ In a study of the prevalence of facial FFA lesions among different ethnicities, the frequency of papules was much higher in the group of Hispanic women (41%) compared with Caucasian women (14%; p = 0.003).^25^ The correlation between clinical type and the presence of papules was described in two Spanish studies, but it showed conflicting results.^21^, ^59^

#### Physiopathogenesis and histopathology

Initially, they were described as a manifestation resulting from inflammation of the facial vellus follicles,^57^, ^58^ but since 2017, with the histopathological description of papules without perifollicular inflammation and the demonstration of the effectiveness of isotretinoin for the treatment of the papules, it is understood that facial papules are an indirect manifestation of skin atrophy associated with the disease.^60^, ^61^, ^62^ Each papule corresponds to a sebaceous gland exceeding the thickness of the dermis thinned by the disease. As histopathology does not always show an increase in the size of the gland because of shrinking during processing, some authors suggest that there is an in vivo dilation of this structure caused by the destruction of the elastic network in the region. Different degrees of inflammation around the sebaceous gland may be present, but should not be considered diagnostic of these lesions, as they are also found in normal facial skin.

This new understanding of the physiopathogenesis of the lesions explains the greater prevalence of papules in males^6^ and in younger individuals,^59^ since these groups have a greater volume of sebaceous glands than females and older individuals, respectively. Likewise, it is understood that treatment of these lesions is not carried out with anti-inflammatory drugs, but rather with retinoids, which will reduce the volume of the sebaceous glands.^60^, ^62^

#### Temporality

There is no data on the temporal relationship between the appearance of papules and the onset of alopecia.

#### Treatment

**Fig. 9:**
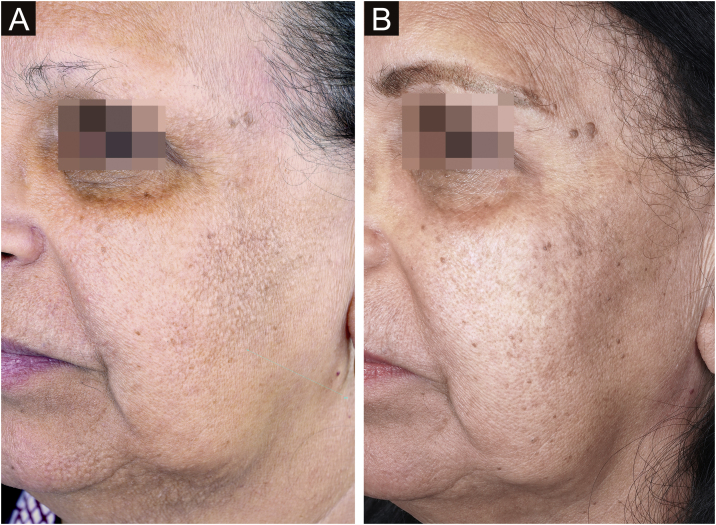
Facial papules in a patient with FFA before treatment with oral isotretinoin in the image on the left and after treatment in the image on the right.

Oral isotretinoin is the treatment of choice to control papules on the face. The dose described in the literature varies from 10 mg per day on alternate days^60^ to 20 mg per day in the first month, followed by 40 mg per day (0.5 mg/kg/day) in the following months (Fig. 9).^61^ The time until lesion improvement is two weeks with the highest daily dose regimen,^63^ while it takes two months to achieve improvement using 10 mg per day on alternate days.^60^ Due to greater tolerability and need for chronic use, smaller doses are ultimately used more often in daily practice.

Some authors prefer the use of topical retinoids for the treatment of papules, due to fear of the risks of systemic medication, but there are no data in the literature proving the effectiveness of these medications in the treatment of facial papules. Moreover, these patients tend to have extremely sensitive skin and the use of any type of topical substances should be done with caution, as there is a proven association of the disease with the use of facial creams.^12^, ^13^

#### Relevance

Facial papules are associated with a greater extension of the disease^6^ and a greater need for the use of systemic medications.^59^

## Final considerations

FFA is a type of cicatricial alopecia with an significant psychological impact on patients^64^ due to the involvement of visible, hairy areas that are difficult to camouflage. The typically slow and asymptomatic progression of FFA often results in delayed patient recognition and diagnosis, sometimes by many years.^65^ The medications that are available to treat the disease are effective in preventing disease progression in most patients, but they cannot reverse what has been already affected at the time of the diagnosis.^6^, ^66^, ^67^ Hair transplantation has good short-term results, but in most cases progressive graft loss occurs over the years .^68^ For all these reasons, early diagnosis of the disease is essential to improve the quality of life of patients with FFA.

Since extra-follicular involvement of FFA may precede the hair loss caused by the disease, the identification of these clinical manifestations can facilitate and anticipate disease diagnosis in many cases. However, there are currently no clinical or histopathological criteria for the diagnosis of FFA through the non-follicular skin changes of the disease, and it is very important to prove primary cicatricial alopecia through a skin biopsy in very early cases. Therefore, the presence of these manifestations should prompt closer surveillance of the scalp with careful assessment of vellus hair density on the anterior border of the scalp, skin biopsy in cases with reduced vellus hairs and photographic follow-up in individuals in whom the density has yet to show any changes.

The understanding of the physiopathogenesis of the disease also changes with the concept that FFA can begin with extra-follicular involvement of the skin. At the same time as it reinforces the possible involvement of substances contained in facial creams, it questions the causal relationship of these products suggested by case-control studies.

Given the relevance of this subject, the authors consider it essential to carry out more studies with appropriate methodologies to characterize the cutaneous involvement of FFA, investigating, when possible, the temporal relationship with the disease follicular involvement. Furthermore, prospective controlled studies are needed to evaluate the effectiveness of currently proposed treatments for extra-follicular manifestations of the disease and their prognostic significance.

## Financial support

None declared.

## Authors' contributions

Aline Donatti: Design and planning of the study and data collection; writing and reviewing of the manuscript; collection, analysis and interpretation of data; critical review of the literature; approval of the final version of the manuscript.

Isabelle I Hue Wu: Design and planning of the study and data collection; writing and reviewing the manuscript; collection, analysis and interpretation of data; critical review of the literature; approval of the final version of the manuscript.

## Conflicts of interest

None declared.
